# Assessing community perspectives of the community based education and service model at Makerere University, Uganda: a qualitative evaluation

**DOI:** 10.1186/1472-698X-11-S1-S6

**Published:** 2011-03-09

**Authors:** Scovia N Mbalinda, Colin M Plover, Gilbert Burnham, Dan Kaye, Andrew Mwanika, Hussein Oria, Isaac Okullo, Wilson Muhwezi, Sara Groves

**Affiliations:** 1 Department of Nursing, School of Health Science, College of Health Sciences, Makerere University, Kampala, Uganda; 2 Department of Dentistry, School of Health Sciences, College of Health Sciences, Makerere University, Kampala, Uganda; 3 Department of Pharmacy, School of Health Sciences, College of Health Sciences, Makerere University, Kampala, Uganda; 4 School of Medicine, College of Health Sciences, Makerere University, Kampala, Uganda; 5 Johns Hopkins School of Nursing, Baltimore, Maryland, 21205, USA; 6 Johns Hopkins School of Public Health, Baltimore, Maryland, 21205, USA

## Abstract

**Background:**

Community partnerships are defined as groups working together with shared goals, responsibilities, and power to improve the community. There is growing evidence that these partnerships contribute to the success and sustainability of community-based education and service programs (COBES), facilitating change in community actions and attitudes. Makerere University College of Health Sciences (MakCHS) is forging itself as a transformational institution in Uganda and the region. The College is motivated to improve the health of Ugandans through innovative responsive teaching, provision of service, and community partnerships. Evaluating the COBES program from the community perspective can assist the College in refining an innovative and useful model that has potential to improve the health of Ugandans.

**Methods:**

A stratified random sample of 11 COBES sites was selected to examine the community’s perception of the program. Key Informant Interviews of 11 site tutors and 33 community members were completed. The data was manually analyzed and themes developed.

**Results:**

Communities stated the students consistently engaged with them with culturally appropriate behaviour. They rated the student’s communication as very good even though translators were frequently needed. Half the community stated they received some feedback from the students, but some communities interpreted any contact after the initial visit as feedback. Communities confirmed and appreciated that the students provided a number of interventions and saw positive changes in health and health seeking behaviours. The community reflected that some programs were more sustainable than others; the projects that needed money to implement were least sustainable. The major challenges from the community included community fatigue, and poor motivation of community leaders to continue to take students without compensation.

**Conclusions:**

Communities hosting Makerere students valued the students’ interventions and the COBES model. They reported witnessing health benefits of fewer cases of disease, increased health seeking behavior and sustainable healthcare programs. The evidence suggests that efforts to standardize objectives, implement structural adjustments, and invest in development of the program would yield even more productive community interactions and a healthcare workforce with public health skills needed to work in rural communities.

## Background

The Ugandan Ministry of Health and key healthcare stakeholders recognize several challenges to the implementation of healthcare systems that both provide community-based primary health and extend services to the entire population. One obstacle preventing many Ugandans from obtaining primary healthcare is the misalignment of population and healthcare workforce distribution. Approximately 88% of the population inhabits rural regions; however, the healthcare workforce predominantly trains and pursues employment in urban settings [1]. The majority of doctors and pharmacists, 70% and 80% respectively, are urban based, serving only 12-16% of the population [2]. The distribution of nurses and midwives is not as dramatically skewed but still unequally distributed with 40% serving the much smaller urban populations [2]. To address this problem, Ugandan healthcare education institutions adopted several strategies to encourage a more equitable distribution of human healthcare resources nationally. One of these strategies, aimed at better meeting the needs of Ugandans in rural settings, was the development and implementation of community-based education and service (COBES) for medical, nursing, dentistry, pharmacology, and radiology students at Makerere University in 2003.

Makerere University College of Health Sciences is forging itself as a transformational institution in Uganda and the region. The College is motivated to improve the health of Ugandans through innovative responsive teaching and provision of service [3]. Other papers have focused on evaluating the COBES program from the perspective of students, faculty, site tutors and alumni [4,5]. This paper focuses on evaluating the COBES program from the perspective of the community, and can assist the College in refining an innovative and useful COBES model that will have even more potential to improve the health of Ugandans. It will help meet the grander challenge of building a health system with the human resource capacity to improve health outcomes in all of Uganda, including the very rural areas.

The COBES model has several objectives. It concentrates on exposing students to the public health and primary health care needs of rural communities early and throughout their education, while fostering interdisciplinary collaboration and self-directed learning. Through these activities, the program aims to instil in students the importance of developing community partnerships, engaging communities as a means to implement sustainable healthcare initiatives, as well as, develop their skills and comfort at accomplishing these goals. Most importantly, however, from a national public health perspective, research supported that these exposures may also encourage students to pursue rural health services [6]. Ultimately, the COBES program both equips students with the practical skills to provide high quality community-based primary care and serve as a tool to redistribute healthcare resources more equitably and effectively throughout Uganda.

Community partnerships are defined as groups working together with shared goals, responsibilities, and power to improve the community [7]. The community partnerships in the COBES model include the community members, other organizations working in the community, and Makerere University represented by the students, site tutors, and Makerere faculty. Community members, as active partners, are involved in assessing, planning, developing, implementing, and evaluating health programs in their own communities. There is growing evidence that this involvement contributes to the success and sustainability of these community-based programs, more easily changing community actions and attitudes. Involving community members as partners and active participants in their own health will have a transformative effect on the involved communities [7,8]. Ndiaye and colleagues pointed out that programs where the community worked side by side with the health workers but were not involved in the program design were not as effective as when there was sharing of power and responsibility that occurs in a true partnership [9]. This approach allows community members to become active participants in solving their own health problems.

This study evaluated, from the perspective of the communities, the COBES model by assessing several key areas: the engagement of the community in identification, implementation and evaluation of the community activities, the effectiveness of the student communication, the value of the health interventions, and the sustainability of the student designed community programs. In addition the community was asked the challenges of having the COBES program in their community.

## Methods

Forty-seven Ugandan community-based sites were annually utilized for MakCHS COBES student placements, and included Community Health Centers at levels III and IV, and regional public and private hospitals. Level III services included continuous basic health prevention and promotion services, curative care with laboratory, and maternity care. Level IV, in addition to Level III services, provided life-saving medical, surgical and obstetrical emergency care [10]. Although the assigned sites provide different levels of primary and curative care, the student experiences in the communities were all similar in that each group partnered with an identified village or community apart from the health centers.

All utilized sites were divided into 4 geographical regions: North (9 sites), East (12 sites), Central (14 sites), West (12 sites). A stratified random sample of 11 sites was selected. The sample was obtained by randomly selecting 3 sites in each of the larger regions and 2 sites in the smaller North region. The sites selected in the North were St Joseph Kitgum Hospital and Arua Hosptial; in the East were Busia Health Center IV, Kumi Hospital, and Atirir Health Center IV; in the Central region were Kiruddu Health Center III, Kayunga Hospital, and Rakai Hospital; and in the West were Nyakibaale Hospital, Rubaare Health Center IV, and Rugazi Health Center IV. For each site there were Key Informant (KI) interviews of the site tutor and community members.

A qualitative approach was selected to allow for an open-ended exploration of the issues. For the interviews, semi-structured guides were developed and used. In March, 2010 the KI tools for both the site tutor and the community informants were pretested in a COBES Level IV health center not selected for the sample. Appropriate changes were made, and the interviewers trained. In late March and April 2010, each of these sites was visited by a team of two people. A deliberate effort was made to match the interview teams for each of the regions with the local languages and customs to ensure ease of comprehension in data collection. KI interviews was conducted with the community- based site tutors who worked for the local site organization and were responsible for the student’s daily activities at the site and the problem based learning that occurred there. The site tutors were supervised by MakCHS for their work with the students, and would know the most recent student community activities. Eleven site tutors were asked how the community activities were selected, to provide a description of these activities, their perception of the community’s interaction with the students, and the sustainability in the community of the students’ intervention. They live in the communities near the health centers, but, as health care providers, may have a different perspective than the community residents with whom the students intervened.

A total of 33 KI interviews were then conducted with 3 community member from each of the sampled site focusing on the areas with the most recent student activities. Informants were selected from the local councils and community members who had interacted with the students. All interviewed at the community level lived in the local community served by the students, and used the local community health facilities. Interviewers obtained information about the community’s perception of the nature of the student community activities, community engagement, student communication with the community, the student intervention project, sustainability of the project, unplanned consequences, and any challenges the community had. Written notes were taken during the interviews by both interviewers with the oral permission of the respondent. The original interviewers transcribed the interview, three members of the team read the information in its entirety, and themes were developed. Summary statements with representative quotes were developed for each theme. The final analysis was shared and discussed among all co-authors, who approved the findings

In addition, one member of the team reviewed the student community reports from the selected sites. Each group of students wrote a group report at the end of the clinical community experience following a prescribed outline from the course syllabi. All the reports from the sampled communities were examined for community activities completed, amount of community involvement noted, project evaluation, and other significant references to the community. These were organized in a chart and shared with the team analyzing the data as background information.

The research was approved by the Institutional Review Boards of the College of Health Sciences, Makerere University and received a waiver from Johns Hopkins University Bloomberg School of Public Health.

## Results

In an effort to provide the community a voice in their experience as partners in COBES the analysis of the data focused on community perception, and the following key themes emerged: community engagement, communication, interventions, sustainability, community impressions, and challenges.

### Community engagement

The philosophy of developing public health interventions with the community through active community engagement and participation lies at the core of the community-based education model. The COBES students were directed to reach out to the community as a means to familiarize themselves with the local culture and involve the community into the students’ interventions. They received specific instruction prior to the experience in the best ways to enter a community to form partnerships. The interviews reflected the efforts students made to connect with the community and how the community received these efforts, offering data affirming that all of the student groups accomplished this objective at their site.

“Home visits were carried out in selected homes and the community needs were asked…the community was actively involved.” (KI, local chairman)

There were, however, variations both in the extent to which the student groups reached out to communities, the means they used to familiarize themselves with the community, and how much they included them in the development of their intervention. The interviews provided insight into the variety of methods students employed to engage their community.

*“The activities included organizing fact finding missions, carrying out focus group discussions and key informant interviews.”* (KI, site tutor)

*“Students asked questions on domestic violence, family planning and major diseases affecting us.”* (KI, community member)

The methods, tools and strategies used by the students were influenced by a number of factors including the goals and objectives of their respective year of study, the site tutor’s guidance, and the cultural protocols of each community. All of the communities reported on how carefully the students listened to them before selecting activities.

*“The students go to communities. Select a topic or a project to work on. Discuss it with the community. Present and discuss their suggestions with the site tutor. The site tutor advises and students make adjustments depending on the suggestions of the site tutor and the community advice.”* (KI, site tutor)

In some instances the students used proxies for community engagement. They substituted home visits and focus groups with community members for key informants such as community mobilizers, a representative of the hospital familiar with community issues.

*“Students come up with their activities and present them to the site tutor. Using community mobilizers to help in selection of the activities because they know the problems in the communities better. The students, site tutor and community mobilizers sit and decide on the community activities to be done.”* (KI, site tutor)

Overall, the community, site tutors, and community mobilizers conveyed that the COBES students made consistent efforts across sites to reach out to the community. Although the students’ methods varied between sites and some did not solicit input from a diverse group of community members, the general trend illustrated that COBES students had community engagement. In the sites in the north, east, and west there were repeated references to more limited community engagement because of language, with none of the students in the group speaking the local language. The central region did not have this problem with more students knowing the local language.

### Communication

To evaluate the quality of the COBES students’ communication, questions were asked that focused on the student’s ability to communicate with communities and the amount of feedback students provided to the community.

### Language

A major theme in the interviews was the communities’ ability to understand the students. There are several different languages spoken throughout Uganda and different strategies were employed to overcome this potential communication barrier. In general the community reported that student groups overcame the language barrier by ensuring that their sub-group divisions had at least one individual who spoke the local language. In the north and west there frequently were no students who knew the local language and they sought the help of translators.

*“All the students who came did not speak Acholi but interpretation was made through teachers from Okwici Primary School. I think they did very well because people understood what was going on.”* (KI, community member in the northern region)

The effectiveness of these efforts to facilitate the students’ community communication was also evaluated.

*“Students’ communication with the community could be ranked as average, since it was their first time. They were respectful to the community members and used mainly English language with translations into Lugbara.”* (KI, local chairman)

Likewise, the manner in which students conducted themselves, the cultural deference they showed, and the respect they exhibited through community interactions facilitated the establishment of strong relationships. Respect for culture was universal across all four regions.

*“Most of them [students] are humble, they speak easily, they use simple and easy to understand language, most questions are easy to understand.”* (KI, community member)

### Feedback

The community interviews revealed that students provided feedback to some communities but not others, that they used written and oral methods to provide feedback, and delivered it to different individuals. The rate of feedback provided across all COBES sites was approximately 50%. There were no regional differences nor was their differences base on level of the health center including the regional hospitals. In one site the community organized tea so the students could give feedback to the whole community. On the other hand, in a different community several of the community members reported that feedback was given but when asked they were unable to verbalize the feedback content. Some of the community members identified feedback as any contact with the students.

*“I think they gave feedback.”* (KI, community member)

*“Yes, I think so, that’s why they came back and distributed mosquito nets.”* (KI, local chairman)

There were, however, site tutors that reported that the students reached out and provided feedback directly to the community. It was not clear if the site tutors actually observed this feedback.

*“Having done research in the community about malaria prevention, the students disseminated findings of their research to community members as one of the community outreach activities.”* (KI, site tutor)

*“With the students, we go back to the communities and give feedback about what the students find out and how they think the community should improve.”* (KI, site tutor)

There was some indication from the community that groups had disseminated information directly to the community

*“It was good that students gave feedback to the local leaders what they had found out and advised the community on what to do next.”* (KI, local chairman)

Several community members reported that feedback was not provided verbally or in a written report. Common responses among these community members were “no” and “none” regarding whether feedback was provided, however, a few individuals elaborated.

*“The students did not give me a written report of their research or what they found in our community. However, they made for us community maps showing each household’s location. So the students did not give a written or verbal feedback because they had little time since they had to go into as many villages as possible.”* (KI, local chairman)

### Interventions

Although a number of activities precede the selection and implementation of community-based interventions, focus is often given to the intervention and outputs because these largely determine how the community is affected. Overall the communities appreciated the interventions and saw positive changes in health seeking behaviour and in levels of general health.

Although the interventions were community specific there were many similarities. In all the regions students provided education and some services in the areas of family planning, how to disclose HIV positive status, malaria prevention, sanitation and hygiene improvement with a focus on hand washing, construction of dish drying racks and latrine covers from local materials, instruction on nutritious ways to use available food, and boiling of drinking water.

Some of the COBES students groups developed interventions moulded by the unique needs they identified in the community.

*“Health education given on latrine usage, bath shelter usage, rubbish pit usage, drinking water safety concerns. Mothers were educated about safe delivery in hospital as opposed to home deliveries. Child immunization education was given in addition to outlining the most prevalent diseases.”* (KI, community member)

Many of them included positive changes in practices at home to prevent disease, promote healthy living, and reduce morbidity.

*“Malaria control was very good for our community, now people don’t spend too much time going to the clinics because of malaria.”* (KI, community member)

*“Currently homesteads are cleaned, bathing shelters and rubbish pits are constructed and clean sources of water are used. Generally speaking malaria and typhoid fever prevention have been emphasized.”* (KI, community member)

Another valuable change reported by the site tutors and the staff from the health center was improved health seeking behavior at the clinics.

*“The patients often tell us that they were told by the COBES students to turn up at the health center every 3 months for de-worming with albendazole. Therefore, the health education has been translated into an improvement in health seeking behavior”* (KI, nurse in charge)

*“The presence of students in communities has so far translated into an increase in patient numbers at the health center. This is because of the health education and the emphasis on the role of seeking care at health facilities. Many people have been enlightened to seek healthcare”* (KI, site tutor)

### Sustainability

The communities reflected that some programs were more sustainable than others as a result of a variety of factors.

*“Yes, these activities are sustainable. The community members have managed to protect against cholera ... no new cases reported in the health facility save for only 2 cases last year.”* (KI, community member)

*“Some of the activities can be sustained. Owing to negligence on part of some community members some of the activities may not be sustained.”* (KI, community member)

The community identified several reasons why the programs had been sustainable including an improved knowledge base and awareness, the development of community skills and leadership, use of easily obtainable materials in the community, and the employment of social structures to ensure continued efforts are maintained. The students in all the districts utilized the local village council leaders, the community mobilizers, the principal and teachers in the local schools, and the church leaders to help them sustain programs. A group in the central region was able to get the hospital to include some of their projects in the hospital plan, further institutionalizing the intervention. The community reported that projects were more likely to be sustained when the local leadership was given written information to help them plan and set priorities on how to continue the program.

*“Community members are able to construct their own latrines and bath shelters with locally available materials.”* (KI, community member)

*“Activities are sustainable; the local leadership can spearhead the continued implementation.”* (KI, community member)

The local village government also played a role in improving the sustainability of the students’ interventions. Local council members and community groups would visit community homes and encourage individuals to adopt health practices advocated by the students. They also established informal rules that would allow them to encourage the changes.

There were several community members who felt that some of the activities were not sustainable, especially those interventions which required additional resources that were not available.

*“No, at Ug shillings 4,000 per net, people still find difficulties in buying mosquito nets. Even some people missed and we would like the students to distribute more.”* (KI, local chairman)

The site tutors were able to provide some perspective beyond the perceptions of the community as to whether the interventions were sustainable.

*“These programs have been maintained and continued in homes. The local leaders formed a committee that moves around homes encouraging them to keep their homes clean and following the steps that students did like clearing bushes and toilet cleaning for some homes like where the person is too old,”* (KI, site tutor)

### Community impressions

General community impressions were very positive, including their overall satisfaction of the COBES program and desire for the students to return.

*“The community appreciated very much the role of COBES students and did learn a lot and they believed that they would learn more if the students kept coming to visit the village. The overall evaluation of the students COBES placements in the community is very good.”* (KI, local chairman)

One site tutor said the community had told him how much they valued the students coming into the community.

*“The community members do appreciate the students very much, especially those that provide health education. For many it is a rare occurrence to be visited by a group of health workers to teach them on basic health promotion and disease prevention principles. So, when they are visited by the students, they cherish such opportunities very much.”* (KI, site tutor)

The overall impressions that the students left on the communities were positive. Despite the challenges the communities expressed that they would appreciate if the student returned in the future.

*“The consequences have been largely positive. The school that students visited when they are here for COBES keep asking me to bring the university students again.”* (KI, community mobilizer)

### Challenges

The community shared their views regarding the challenges of implementing the COBES program in their community. Some student community health education activities were hindered by low community literacy rates and the student’s inability to reinforce themes over a more extended period of time.

*“Community people often forget the themes of the [health] talks. They only get reminded when the students come back.”* (KI, local chairman)

Community fatigue and the need to incorporate community incentives were also commonly expressed issues with the COBES program. Many communities felt like they were a teaching laboratory for the students. Some communities were used repeatedly, with first year students doing the same assessment, asking the same questions, and making the same community diagnosis year after year. These communities wanted more coordinated interventions that would help the community grow in health.

The site tutors said the community sometimes felt that they should receive something for their time and participation. Site tutors reported that this was mentions by communities that hosted many students in one year. Community leaders and community mobilizers also felt they should receive remuneration for their additional time, especially since other schools that used their site for community based education provided it.

*“The community expects something tangible, out of their goodwill and willingness to interact with the students. The students often do not have guidelines on what is expected of them in the community. The tutors from the university who are supposed to guide us and guide the students neglect these students a lot.”* (KI, site tutor)

*“Limited time, costs, lack of transport, lack of motivation for the community leaders who take students to the communities, lack of incentives for the participating communities like giving gloves and cotton to the TBAs are incentives to motivate them to participate and allow students to ask them questions.”* (KI, site tutor)

## Discussion

The community felt that the COBES model had significant impact in decreasing disease, increasing health knowledge, encouraging health seeking behaviour, and increasing primary prevention and primary health care. As MakCHS students learn to implement this model in real service learning, they are poised to create change in the management of primary health care in rural Uganda. The students learn how to provide optimum service delivery, and how to work with the community to create positive change. This innovative teaching positions MakCHS to have a large and a different impact on the health system in Uganda and internationally than the traditional curative medicine curricula.

The evaluation of this program from the perspective of the community, linked with the perspective of the alumni, faculty, and current students [4,5], allows the COBES program to build on the many positive components, and improve the model to better serve the community within a constructive and inventive teaching model.

The community appreciated the students’ involvements in their community. They were very humbled by the students’ willingness to come into their home, and sit and talk with them. They reported there were occasional outreach programs, health professional did come to their communities, but it was rare for them to make home visits. They particularly appreciated the health education they received from the students. As a result, several of the communities and the health workers in the community commented on a decrease in major communicable diseases, specifically malaria, typhoid, cholera, and diarrheal diseases. Other community members mentioned that when the students were present they had better service at the local clinics with marked decrease in waiting time. In summary they reported that as a result of student community interventions there was better health seeking on the part of the community members. The community perceived that the MakCHS COBES approach was the right approach to have, creating opportunities for better health in rural Uganda

Although the community was pleased with the student involvement they said they would like more investment in the community to create more sustainable programs. They would like the students to build on their practices, not always asking the same questions or start at the beginning with the same assessment each year. Students could benefit from participating in programs that originated with their fellow students’ effort, and help them develop and grow over time to better meet needs. The community wanted to have more of a partnership and to have some motivation or incentives to continue to improve their community.

The inclusion of community members in the assessment, development and execution of public health interventions serves as the foundation for developing interactive community partnerships and lies at the core of community-based education. Integrating the community in this way helps build trusting relationships and enables program developers to better understand cultural nuances, gain perspective into the needs and opinions of the community and serve as a guide to cater interventions to accommodate unique community identities [11-14]. Community engagement acts as a powerful vehicle for bringing about environmental and behavior change to improve the health of communities and their members.

The COBES students effectively employed several methods to reach out to the community and include them in the development of their interventions. Students held community meetings and focus groups and reached out to community leaders to familiarize themselves with the local perspectives. There were, however, no examples from the interviews that the students specifically partnered with the community to mutually decide on the program interventions. The students collected data from the community, discussed their impressions with site tutors and health staff, but many missed an important step of sharing their data with the community as a group, asking for their input before deciding on the project for implementation.

There are several ways the COBES program can work to address this inconsistency. One involves the development and implementation of a document which outlines the students’ responsibilities related to community engagement with a structured guide presenting methods to connect with communities. The second requires appropriate training of site tutors which impresses the importance of encouraging, educating and facilitating student community partnership.

Integrally linked to community partnership and essential to successful community-based education, is the quality of communication between the partners. Effective communication can help build trusting relationships, provide vital information during assessments to guide care, improve understanding and adherence to recommendations and serve as a tool to evaluate quality, allowing for modification of interventions in vivo and in the future [15-18]. The two elements related to communication assessed through the interviews were language barriers and the nature and consistency with which students provided feedback to communities.

Across sites, the students and community were able to successfully overcome the challenge of language barriers where they existed and communicate effectively. The resilience of the community and students, and coordination with community translational resources resulted in an environment where the communities were able to provide information and receive public health education effectively. Community members said that they understood the students’ messages. The community acknowledged that the students were mindful of the local culture and were able to more easily connect with the community because they were humble and respectful. In general, the community and students communicated.

The translators were a valuable communication tool; however, it could be more beneficial to the community to match student language skills with the communities in which they will work. The community would also benefit from students with knowledge of the local customs and culture. With more free flowing communication the students would gain skills in how to work with communication issues like low literacy and marginal knowledge of health and healthy activities.

Quality communication, within the context of community-based education, requires continuity. A dialogue with the community should begin before an intervention is initiated and continued after its completion. Providing communities with feedback is an effective way to maintain continuity of communication. The communication of findings, accomplishments, and challenges also serves as a tool to engage the community in partnership integrating them into the entire process. The development of standardized protocols for the students could serve as a tool to improve the rates and quality of feedback and communication.

The students’ community assessments were instrumental in determining the interventions they chose and revealed each community’s unique health needs. It was not clear if the community participated in the selection of the interventions chosen by the students, or if the community even saw a need for these interventions selected. This is necessary step that needs to be added to the COBES curriculum.

A fundamental measurement of an intervention’s quality is the degree to which it can be sustained. The sustained operation of public health programming increases the scope of its impact and, in some cases, is required for an intervention to witness any substantial health benefits [19-22]. The COBES students were directed to select projects that integrated strategies enabling communities to maintain the interventions after they left. In many communities the COBES students succeeded in accomplishing their goal of implementing programming and providing education that the community believed translated into sustainable public health change. Community leaders encouraged community members to maintain the health practices that the students demonstrated to the communities. Likewise, several months after students left, site tutors reported that homes were still clean, community members were still equipped and utilizing built latrines and bath shelters, and engaging in activities such as clearing brush and managing stagnant water sources where mosquitoes bred. The way that the students provided education to the communities, first by demonstration and then actively engaging the community to participate, improved the sustainability by empowering community members.

Although the education and interventions that the students implemented were usually identified as sustainable, they should continue to coordinate with community mobilizers, site tutors, and community members to ensure that all interventions have a sustainable plan and community support to continue after the students leave.

The development and maintenance of community-based interventions requires the management of numerous challenges. Conducting community assessments and coordinating the implementation of interventions in diverse communities requires cultural sensitivity, the coordination of activities with key stakeholders, the appropriate investment and acquisition of resources and systems to evaluate productivity and quality [23,24].

The community also faced several challenges including language barriers, community protocols and community fatigue. The coordination of the program should be sensitive to language and include at least one student who speaks the local language and is familiar with local customs. This will facilitate entry into the community, increase the effectiveness of health education, and show respect for the community. This teaches students the importance of culture and language in the provision of quality health care, and provides them with an opportunity to experience it first hand.

The communities also identified community fatigue as an issue in the interviews. The community members and site tutors reflected that hosting students throughout the year and providing them with their time, resources and interviews, frequently without any tangible benefits, was troubling and discouraged willingness to participate. The community members talked about the importance of incentives to make their participation more worthwhile. To meet this challenge site tutors, faculty and students will have to be more creative with which communities they reach out to in each region and the methods they use to avoid overwhelming or inconveniencing communities. The University could look at the potential of a pilot project, suggested by the community, which could grow a current student initiated intervention program to increase the depth and broaden the approach, rather than the students always starting over again.

Developing more creative educational methods, providing materials that are appropriately catered to community members’ literacy levels and investing in the community’s ability to sustain the education that students provide will also allow for students to rotate to other communities to prevent community fatigue. Efforts to improve the program for the future will require attention to the shortcomings identified by the communities, and increased skills training for both the community and the students in partnering.

Limitation of the study included variability in the interview styles of the teams that were sent to each region resulting in the collection of varyingly robust data. Some of the interviewers probed the community members they interviewed collecting answers rich in description, whereas, other interviewers provided less. This diminished both the amount of data and the ability to develop strong comparisons between regions. These were Key Informant Interviews and do not lend themselves to quantitative analysis. There was no comparative quantitative community health data before and after COBES was introduced, nor between COBES sites.

## Conclusions

Communities hosting Makerere students reflected that they valued the students’ interventions and the COBES model. They reported witnessing a variety of health benefits including fewer cases of disease, increased health seeking behavior and the implementation of sustainable healthcare programs. This is an innovative program that teaches MakCHS students the value of community partnership and implementing well planned community programs. Although challenges existed which compromised the full potential of the program, such as community fatigue and lack of resources, the communities overwhelmingly expressed a desire to have the students return. The evidence suggests that efforts to standardize objectives, implement structural adjustments and invest in the development of the program will yield even more productive and efficient community-based interventions and a healthcare workforce equipped with the public health skills to work in rural settings.

## List of abbreviations used

COBES: community-based education and service; MakCHS: Makerere University College of Health Sciences; KI: Key Informant.

## Competing interests

The authors declare that they have no competing interests.

## Authors' contributions

SM and SG were involved in the conceptualization of the assessment, analysis and interpretation of the results, and in contributing to the writing of the manuscript. CP assisted with the analysis, interpretation, and wrote the manuscript. GB was involved in the conceptualization of the assessment and the interpretation of results and preparation of the paper. DK, AM, HO, IO, and WM were involved in the conceptualization of the assessment and contributed to the review of the manuscript.
